# The Role of Gut Microbiota in Some Liver Diseases: From an Immunological Perspective

**DOI:** 10.3389/fimmu.2022.923599

**Published:** 2022-07-13

**Authors:** Li Wang, Zheng-Min Cao, Li-Li Zhang, Juan-mei Li, Wen-liang Lv

**Affiliations:** Department of Infection, Guang’anmen Hospital, China Academy of Chinese Medical Sciences, Beijing, China

**Keywords:** gut microbiota, liver diseases, immunity, metabolism, review

## Abstract

Gut microbiota is a microecosystem composed of various microorganisms. It plays an important role in human metabolism, and its metabolites affect different tissues and organs. Intestinal flora maintains the intestinal mucosal barrier and interacts with the immune system. The liver is closely linked to the intestine by the gut-liver axis. As the first organ that comes into contact with blood from the intestine, the liver will be deeply influenced by the gut microbiota and its metabolites, and the intestinal leakage and the imbalance of the flora are the trigger of the pathological reaction of the liver. In this paper, we discuss the role of gut microbiota and its metabolites in the pathogenesis and development of autoimmune liver diseases((including autoimmune hepatitis, primary biliary cirrhosis, primary sclerosing cholangitis), metabolic liver disease such as non-alcoholic fatty liver disease, cirrhosisits and its complications, and liver cancer from the perspective of immune mechanism. And the recent progress in the treatment of these diseases was reviewed from the perspective of gut microbiota.

## Gut Microbiota and Immunity

### Interactions Between Gut Microbiota and the Immune System

The human intestinal flora mainly includes bacteria, viruses, fungi and archaea (1), and the midst carries at hand 1.5 kilograms of symbiotic bacteria (2). The number of bacteria in the intestinal tract is huge (about 10^14^ in total), and there are more than 2,000 species in abundance. The total number of genes is 100-400 times than that of human beings (3). In these microbiota, the research mainly focuses on bacteria. Bacteria are mainly composed of obligate anaerobe, facultative anaerobe and aerobic bacteria, and the six main phyla of healthy adults are Actinobacteria, Firmicutes, Bacteroidetes, Proteobacteria, Clostridium and verruciformes, the former two accounting for more than 90% of the total (4). Intestinal flora plays a variety of roles in intestinal barrier, nutrition, metabolism and immunity by mediating various forms of host response. The microbiome has been proved to promote the maturation of immune cells and the normal development of immune function (5). Bouskra et al. (6) showed that specific intestinal symbiotic bacteria could promote the development of solitary lymphoid follicles, in which Gram-negative bacteria played a decisive role. Besides, the metabolites of gut bacteria affect the number of immune cells (7).

Innate immune cells distribute in the mucous membrane at the host-microbe interface, which is the first line to detect microbial components or productions and transmit signals to the host. Microbial associated molecular patterns (MAMPs) expressed by intestinal flora activate pattern recognition receptors(PRRs) such as innate immune cells binding toll-like receptors (TLRs) to promote intestinal immune tolerance. When the flora is disturbed, MAMPs stimulate macrophages and dendritic cells to generate pro-inflammatory cytokines, leading to immune imbalance. Innate lymphoid cells(ILCs) are the lymphocyte population enriched in mucous membrane. It was found that the secretion of IFN-γ by ILCs can affect the composition and distribution of intestinal flora (8).

Intestinal specific immunity is provided by the mucosal immune system. T cells and B cells in the mucosa and secretory immunoglobulin A(sIgA) secreted into the intestinal lumen jointly complete the local intestinal mucosal immune function (9). Intestinal bacteria also influence adaptive immune responses, mainly the development and differentiation of cluster of CD4+ and CD8+ T cells. The number of CD4+ T cells and CD8+ T cells in intestinal tract decreased, and the number of IL-17-producing T helper(Th17) cells and regulatory T(Treg) cells in intestinal tract decreased to varying degrees (10). Also, many experiments (9, 11–13) have confirmed the intestinal flora could affect the accumulation and functional maturation of Treg cells in the colon. Besides, researches have shown that intestinal flora can induce intestinal epithelial cells(IECs) and mononuclear macrophages to secrete cytokines, thus promoting IgA type conversion and preventing pathogen infection (14).

### Intestinal Mucosal Barrier

**Figure 1:**
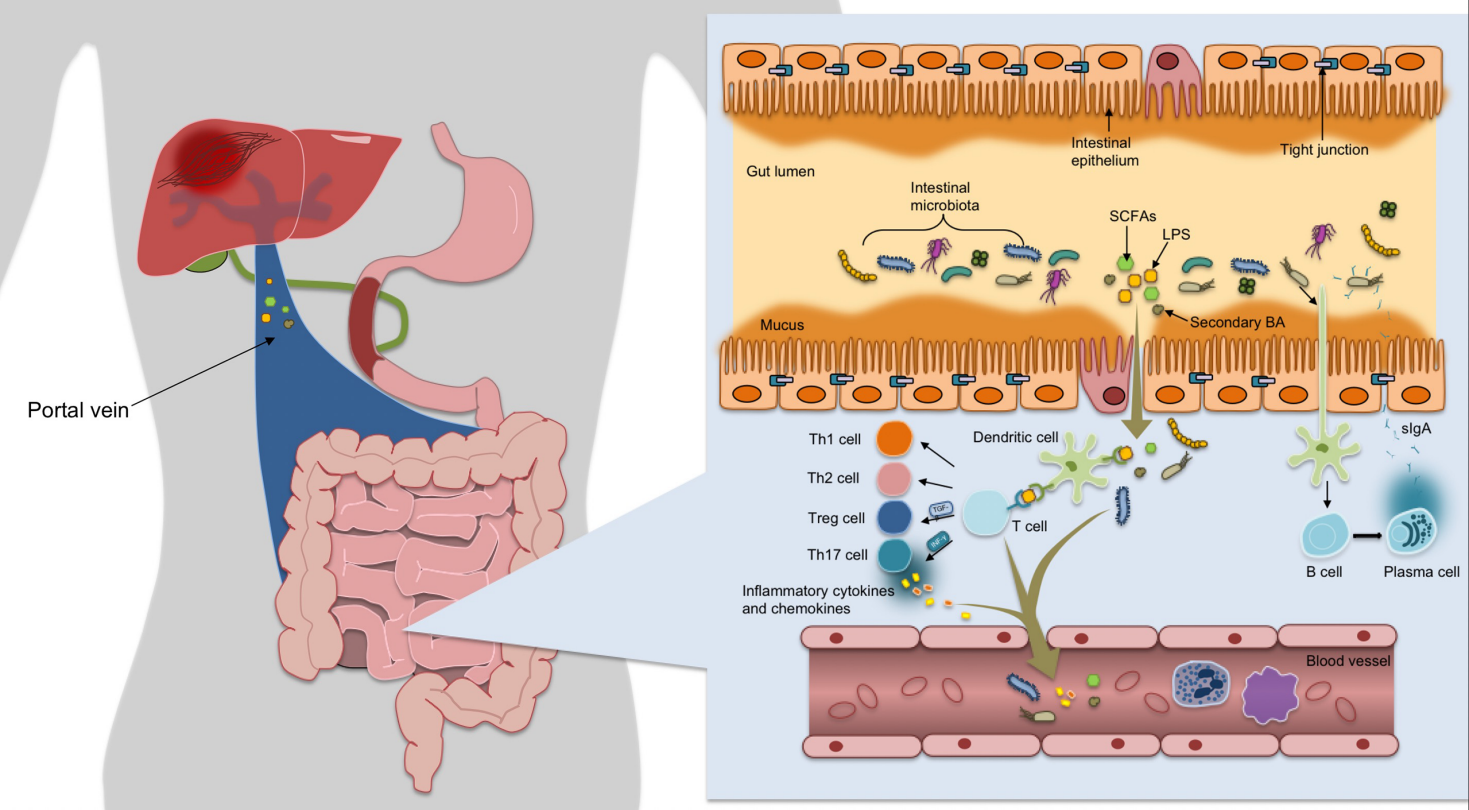
The mechanical barrier is mainly composed of mucus, IECs and TJs between cells, which can maintain normal permeability of intestine, and prevent intestinal bacteria, antigens and other substances from entering the lamina propria. After ingesting, processing and presenting exogenous antigens, the immune response is coordinated by secreting cytokines and producing antibodies, further activating T and B lymphocytes to establish an effective adaptive immune response and inducing mucosal immune response or immune tolerance. Once bound to luminal antigens, DC pattern recognition receptors express co-stimulatory molecules and cytokines involved in regulating the differentiation of Th cells and Treg cells into CD4+ T cells, maintaining Treg/Th17 balance and forming immune homeostasis. The gut microbiota promotes the differentiation of IgA-secreting plasma cells by activating DC cells to secret B cell activating factor, thereby releasing sIgA to encapsulate bacteria to form a complex.

Intestinal mucosal barrier function acts as a defense against the outside world through mechanical barrier, chemical barrier, immune barrier and microbial barrier (**Figure 1**). The mechanical barrier is mainly composed of mucus, IECs and tight junctions(TJs) between cells (15). Mechanical barrier can maintain the integrity of epithelial cell structure and biological function, maintain normal intestinal permeability and prevent intestinal bacteria, antigens and other substances from entering the lamina propria (16). The chemical barrier of intestinal mucosa is formed by the interaction of gastric acid, bile, various digestive enzymes, lysozyme and antimicrobial proteins(AMPs). Human intestinal AMPs mainly include defensin, C-type lectin, lysozyme, Cathelicidins, phospholipase A2, angiopoietin 4, and so on. For instance, α-defensin 5 was found to be a key factor in regulating intestinal microbiome composition (17). RegIIIγ can directly combine with peptidoglycan to kill Gram-positive bacteria, and has a variety of functions such as antibacterial, anti-inflammatory, injury repair and promoting benign proliferation of epidermal cells (18). Cap18 and Cap11, members of the Cathelidicin family, have strong antimicrobial activity, especially against Gram-negative bacterio-pathogenic microorganisms (19). In a word, AMPs can resist pathogenic microorganisms, fight inflammation, regulate immunity, regulate intestinal flora, promote injury repair and cell proliferation, etc. Intestinal mucosal immune barrier includes innate immunity and acquired immunity, in which innate immunity refers to mucins and AMPs secreted by IECs, while acquired immunity mainly includes gut-associated lymphoid tissue(GALT) and sIgA. GALT is the largest lymphoid tissue in the body. After ingesting, processing and presenting external antigens, it can coordinate the immune response by secreting cytokines and producing antibodies, further activate T and B lymphocytes to establish an effective adaptive immune response, and induce mucosal immune response or immune tolerance (20). SIgA, released by B cells, wraps around bacteria to form a complex that stimulates intestinal mucus secretion, prevents bacterial adhesion to intestinal mucosa and neutralizes toxins produced by bacteria. It can also regulate the composition of microflora and help to maintain the balance of the environment *in vivo (* 21). In order to avoid harmful immune response, host and gut microbiota have gradually established a mutually beneficial relationship through mutual evolution and adaptation through two-way communication. Thus, gut microbiota and intestinal mucosa interact and depend on each other to form a microecosystem, namely intestinal mucosal microbiota barrier. Under normal conditions, the abundance and diversity of intestinal flora are in dynamic balance, which can prevent the invasion and colonization of pathogens, and are very important for maintaining the intestinal mucosal barrier function (22). However, the imbalance in microbial population and function, or dysbiosis, leads to disruption of TJs in the gut barrier; this morphological change results in the increase of gut permeability, where bacteria or their increased gateway for products will enter the liver (23). At the same time, it can also cause metabolic disorders.

### Composition and Metabolites of Intestinal Microorganisms

Lipopolysaccharide(LPS) and lipid teichoic acid(LTA) are the membranes of the cytoderm of Gram-negative bacteria and Gram-positive bacteria in the intestinal tract, respectively. Under normal circumstances, intestines are high in LPS and LTA. If LPS and LTA continuously stimulate the innate immune system of the intestinal tract to produce inflammatory response, it will cause very serious immune diseases (24). Peptidoglycan is the main component of bacterial cell wall. Studies have shown that peptidoglycan mainly affects the innate immune system through PRRs nucleotide oligomerization domain(NOD)1/NOD2 (25). Flagellin has traditionally been thought of a virulence factor that contributes to host cell adhesion and invasion, but it has now become an effective immune activator, shaping innate and adaptive immunity during microbial infection (26).

Gut microbiota can produce metabolites, such as short-chain fatty acids (SCFAs), trimethylamine oxide (TMAO), bile acids(BAs), tryptophan and its metabolites, pyruvate and lactic acid, through food degradation, biotransformation and secretion. Dietary fiber is fermented through gut microbiota to produce SCFAs. 90%~95% of SCFAs in intestinal tract are acetic acid, butyric acid, propionic acid (27). As the energy source of IECs, SCFAs can affect the gene expression necessary for intestinal epithelial barrier and defense function, and can regulate natural and specific immune cells (28). Acetic acid binds to G protein-coupled receptor(GPR)43 of dendritic cells (DCs) and induces B cells to produce infection-free globules A (IgA). Propionate and butyrate can inhibit the expression of CD40 in bone marrow dendritic cells (BMDCS), reduce the production of IL-6 and IL-12P40, and thus inhibit the activation of BMDC (29). Butyrate can affect Treg differentiation by acting on GPRs (30). TMAO is an indirect product of gut microbiota metabolism, which can promote the development of angiocardiopathy by inducing inflammation and stress response (31). Tryptophan(Trp) maintains the balance between gut immune tolerance and intestinal flora maintenance. Intestinal bacteria with Trp enzyme can metabolize Trp to produce indole and its derivatives, such as Coryella generalis, Bacteroidetes, etc.

## Gut-Liver Axis

Since Marshall proposed the concept of gut-liver axis in 1998, intestinal relations with liver disease more and more get the attention of people (32). The gut-liver axis consists of lymphatic system, the portal vein, and bile circulatory system. Intestinal tract and liver interact closely with each other through substance metabolism, immune regulation and neuroendocrine system, and form a complex network structure (33). In terms of embryonic origin, both intestinal and hepatic organs originate from the same foregut, and precursors of enteric-associated lymphocytes originate from the developing liver. In physiology and anatomy, the two organs of intestine and liver are interconnected through the portal vein system. 70% ~ 80% of the blood flow of the portal vein is diverted to the liver, transmitting a variety of signals such as nutrients, genetics and environment (23). The two organs are characterized by lymphocytes that share homing and recruitment pathways. The liver is one of the organs most exposed to gut bacteria and their metabolites (34). Enteric-derived T lymphocytes may also cause inflammation of the hepatobiliary tract. Bacteroides, Eubacter, bifidobacterium, Lactobacillus and so on in the intestinal mucosa constitute the biological barrier, which can inhibit the value of pathogenic microorganisms and protect the host from the invasion of exogenous microbes. The intestinal barrier provides the first line of defense against exposure to exogenous substances in the gut. The liver is the second line of defense against antigenic and inflammatory vectors that escape the intestinal barrier.

**Figure 2:**
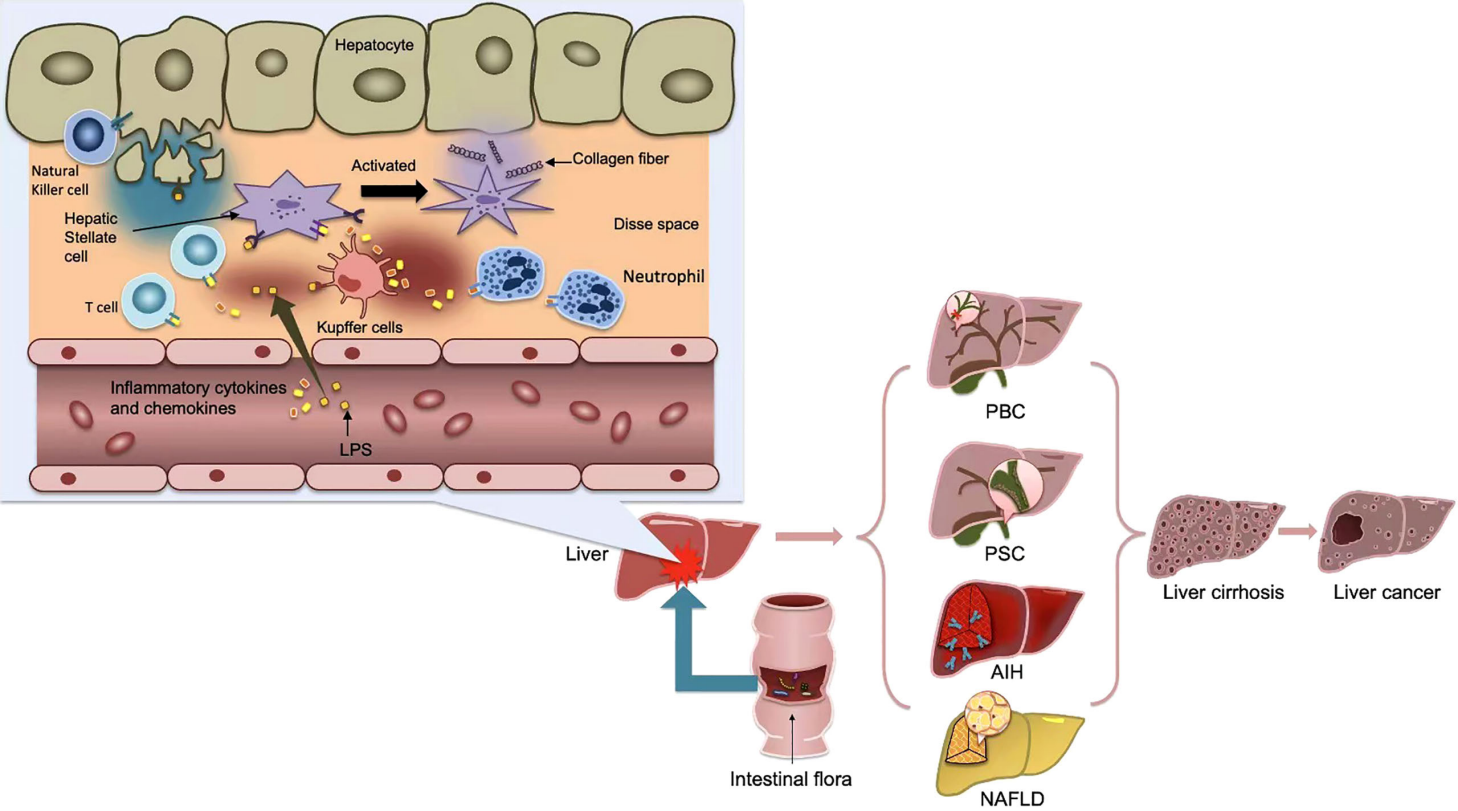
After the activation of the adaptive immune system, some bacterial products, such as LPS, or inflammatory cytokines and chemokines produced, flow into the portal vein system through the upper and lower mesenteric veins, and finally into the liver, causing the activation of kupffer cells and hepatic stellate cells(HSCs), thus further causing the occurrence of liver inflammation and fibrosis. Metabolic products of gut microbiota cause inflammation in liver tissue and affect liver metabolism, thus promoting the occurrence of various liver diseases, and eventually develop into cirrhosis and even liver cancer.

Normally, bacteria and their metabolites, toxins and other harmful substances can be blocked by TJs in the intestinal epithelium. However, once the natural barrier is breached, it is recognized by dendritic cells(DCs) or activated by the adaptive immune system *via* Treg cells and activates the nuclear factor-κB (NF-κB) by TLR and Nod-like receptor(NLR) *via* the pathogen-associated molecular pattern(PAMP). The study confirmed that the expression of TJ-related proteins occludin and Claudin-1 was significantly reduced, leading to increased intestinal permeability and endotoxemia (35). The resulting inflammatory cytokines and chemokines flow through the upper and lower mesenteric veins into the portal vein system, and finally into the liver (36). On the one hand, the liver will be directly damaged and Kupffer cells will be activated, and the related inflammatory factors further can cause intestinal mucosa injury (**Figure 2**). On the other hand, the decreased phagocytosis ability of Kupffer cells and hemodynamic that has changed in liver cirrhosis, will cause functional disorders of intestinal secretion, absorption, barrier and circulation, and eventually increase intestinal barrier energy damage (37). The progression of chronic liver injury was accompanied by loss of tight junction proteins such as Claudins3, 5, and 7 (38). The secretion of TNF-α was significantly increased in patients with cirrhosis, which resulted in abnormal TJ quantity and function (39). In the decompensated stage of cirrhosis, the activated intestinal macrophages participate in disruption of intestinal epithelial barrier by secreting TJ regulators such as NO and IL-6 (40). Damage to the intestinal barrier further will affect liver repair, thus creat a vicious cycle.

The synthesis and secretion of BAs in liver and their reabsorption in the intestine form the gut-liver cycle. BAs are produced by cholesterol in liver and metabolized in gut by gut microbiota. About 90% to 95% of BAs are absorbed at the far end of the distal ileum and then transported to the liver, where they recombine with taurine (in mice) or glycine (in humans) to form bile salts. About 5% to 10% of BAs are degraded and biotransformed by microorganisms mainly in the intestinal tract, and some of them are excreted by feces. The transformation of BAs in the intestinal tract is mainly accomplished by Bacteroidetes, Eulobacter and Clostridium in the intestinal anaerobe. Taurine and glycine are decombined with bile salts by the action of bile saline hydrolysis enzyme to form separate free BAs (41). The modification of BAs by microorganisms not only changes the signaling pathway of BA receptors, but also changes the composition of the microbiome, thus affecting the metabolism of the host (42). BAs are not only involved in the digestion and metabolism of nutrients, but also act as signal molecules and metabolic regulatory factors, activating the signaling pathways of nuclear receptor and G-protein-coupled receptor (GPCR), regulating liver lipid, glucose and energy balance, and maintaining metabolic balance in the body (43, 44).

## Gut Microbiota and Some Liver Diseases

### Autoimmune Hepatitis

Autoimmune hepatitis(AIH) is an abnormal immune reactivity-mediated intrahepatic inflammatory disease targeting hepatocytes, characterized by varying levels of elevated serum transaminase, positive characteristic autoantibodies, hypergammaglobulinemia, and characteristic changes in liver histology, and usually responds well to immunosuppressive therapy (45). The disease occurs mostly in women, with a global incidence of about 0.09% and an increasing trend year by year (46). AIH is caused by the destruction of patients’ autoimmune tolerance, and its etiology and pathogenesis are not fully understood. Currently, AIH is generally believed to be the result of genetic susceptibility interactions, molecular simulation, autoantigen response, immune regulatory dysfunction, gut microbiota and other factors.

Through mouse experiments, scholars have found that intestinal microbe flora maladjustment and barrier function obstacle, inflammation response and systemic autoimmunity marker were concerned (47). The imbalance of microbial communities was thought to be associated with abnormal immune responses (48). The mechanism may be related to the production and signal transduction of SCFAs, intestinal nucleoside signal transduction and so on (49). One study identified the changes of the structure and function of fecal flora in patients with AIH, suggesting the potential of intestinal flora as a non-invasive biomarker for disease stratification in AIH (50). They indicated that the abundance of Veillonella, Streptococcus, Lactobacillus and Klebsiella was increased in AIH compared with healthy controls(HCs) (50). In another study in Africa, Blautia, Faecalibacterium, Haemophilus, Bacteroides, Streptococcus, Eubacterium, Butyricicoccus, Veillonella, and Lachnospiraceae were reported abundant in AIH (51). Veillonella, Lachnospiraceae, Bacteroides, Ruminococcaceae and Roseburia, were selected as the AIH microbial biomarkers (52). In summary, most researches have confirmed the overproportion of Veillonella in the intestinal flora of AIH patients. In general, fecal microbiome diversity is declining in most AIH patients (50, 53, 54). Genetic factors of the major histocompatibility complex(MHC) have been identified as triggers of AIH, with human leukocyte antigen DR 3(HLA-DR3) and DR4 being the most commonly recognized (55, 56). One study showed that intestinal microbiota was significantly altered in HLA-DR3^+^ AIH models, but not in wild-type mice, suggesting that genetic factors may induce changes in the mouse microbiota and are related to AIH susceptibility (57). In conclusion, environmental factors and specific genotypes leading to specific microbial communities may contribute to susceptibility to AIH. Immunohistochemical analysis of AIH patients showed decreased expression of TJ protein and increased serum LPS, suggesting that the integrity of TJ was impaired and there may be bacterial translocation (58). Manfredo et al. found that enterococci could migrate to organs and tissues such as liver, spleen, mesentery, and mesentery lymph nodes, resulting in autoimmune diseases(AIDs) such as AIH (59). Bacterial translocation is also an important factor in liver injury in AIH patients. Concanavalin A (ConA) induced acute hepatitis in mice is a widely used animal model to study AIH, but ConA could not induce hepatitis in germ-free mice, while ConA in mice carrying pathogenic Salmonella and Streptococcus could induce severe liver injury (60). Study indicated that this was related to the increase and enhanced activity of intestinal DCs induced by bacteria (60). Subsequently, DCs lead to the activation of natural killer cell(NKT), which can secrete a variety of cytokines, activate other immune cells, aggravate inflammatory response or directly lead to liver damage (60). Centa et al. found that TLR-mediated microbiota induction in liver hematopoietic cells and intestinal flora contributed to AIH in mice (61).

The standard treatment for AIH is immunosuppressive therapy (the combination of glucocorticoid and azathioprine). How to avoid or minimize the side effects caused by long-term treatment is the focus of clinicians. Probiotics can not only restore the composition of the intestinal flora, but also have the function of regulating the immune system. Probiotics are safe and effective in alleviating AIDs and are expected to be adjunctive or even alternative therapies. Some researchers gave AIH mice intragastric administration of compound probiotics, and found that it could inhibit the infiltration of inflammatory cells in the liver, reduce Th17 and Th1 cells and serum transaminase, and reduce AIH (62). Moreover, Treg cells rised in probiotic group, indicating that compound probiotics have immunomotor effects (62). The mechanism is that compound probiotics block the translocation of potentially harmful substances of intestinal origin to the liver. As a result of this blockade, intestinal LPS-activated TLR4/NF-κB pathway is inhibited, resulting in reduced production of inflammatory factors and promoting remission of AIH. Bifidobacterium lactate 420 had good function in alleviating experimental AIH (63). Both Bifidobacterium longum LC67 and Lactobacillus plantarum LC27 can inhibit inflammation by inhibiting the NF-κB inflammatory pathway (64). Wang et al. found that a special mouse intestinal strain Bacteroides acidifaciens could reduce liver cell apoptosis in a CD95-dependent manner, thereby improving liver injury. The determination of Bacteroides acidifaciens function may provide an opportunity for its future use in the treatment of liver diseases (65). Prebiotics are functional substances that can improve the health of the host. They are not digested and absorbed by the host, but promote the reproduction and metabolism of intestinal probiotics. Inulin is an indigestible, biodegradable natural functional dietary fiber and a natural prebiotic. Yamaguchi et al. found that liver damage was significantly reduced histologically and serologically in mice fed inulin for 12h after ConA administration (66). In addition, antibiotics or specific vaccines can effectively inhibit the growth of enterococci and control the disease progression (59).

### Primary Biliary Cholangitis

Primary biliary cholangitis(PBC) is a chronic intrahepatic cholestasis with the most common clinical manifestations of fatigue and itchy skin, which can lead to liver failure or even death (67). PBC features chronic nonsuppurative destructive small cholangitis. The E2 component of pyruvate dehydrogenase complex (PDC-E2), which is the main mitochondrial antigen of human body, may stimulate the production of anti-mitochondrial antibody(AMA) and induce PBC (68, 69). Serum AMA is the most valuable laboratory indicator for diagnosis, and AMA-M2 in particular is highly specific. Both genetic variations and environmental factors increase susceptibility to PBC. Biliary epithelial cells are generally considered as passive targets of immune attack in PBC patients (70). However, bile duct dedifferentiation, stress, senescence and DNA damage may promote PBC (70).

Gut microbiota can affect the pathogenesis and progression of PBC at multiple levels. Some scholars have found that fecal microbiota is associated with fibrosis and cirrhosis of PBC, and a decrease in alpha diversity and an increase in Weisella in advanced fibrosis community (71). The microbial diversity of PBC individuals decreased significantly (54). Furukawa et al. found that contrasted with healthy persons, PBC patients had lower bacterial diversity, higher abundance of Lactobacilli, and lower abundance of beneficial symbiotic clostridium (72). A Chinese study showed that there were more rich genera in PBC, including Veillonella, Haemophilus, Streptococcus, and so on (73). Evidence suggests that intestinal dysbiosis and excessive toxic BAs play key roles in the nosogenesis of PBC (74, 75). The gut microbiome affects BA signaling by metabolizing BAs through specific enzymes (76). BAs participate in multiple signaling pathways, including immune homeostasis, metabolism, and fibrosis by acting on farnoid X receptor(FXR) and TGR5 (76, 77). Molecular simulations between microbial and host-derived antigens may then initiate or enhance autoimmune responses in genetically susceptible individuals. Berg et al. found that molecular mimicry between mycoplasma surface molecules and autoantigen epitopes presumably played an important role in the etiology and pathology of PBC (78). Bogdanos et al. found that the cross-reaction of IgG3 antibodies in beta-galactosidase of Lactobacillus delbrueckii with major mitochondrial autoepitope was characteristic of PBC (79).

Ursodeoxycholic acid(UDCA), a first-line treatment for PBC, is efficient in about two-thirds of patients with early PBC (74, 80). The dynamics of BAs also have an effect on gut microbes. The secondary BAs levels were negatively correlated with PBC-enriched intestinal flora (81). UDCA partially reversed intestinal microbiome disorders in PBC patients and reversed abundance of 6 related bacterial genera after 6 months of treatment (54). The prognosis of PBC is related to the intestinal flora. Han et al. (82) found that after 12 months of UCDA remedy, TB levels in PBC patients were associated with a unique gut microbiome. One of the pharmacological effects of BA sequestrant is to increase the activity of the BA receptor TGR5 in the gut, leading to the release of the downstream hormone glycoadenotide peptide -1 (GLP-1) (83, 84). The Lachnospiraceae species are known to produce SCFA, which is generally considered to be one of the most vital immunomodulatory molecules of microbiome metabolism (85). Klebsiella pneumoniae has been found to be a pro-inflammatory pathogen that causes intestinal barrier damage and Th17 cellular immune responses in PSC (86). Li et al. treated PBC patients with BA sequestrant cholestyramine

as increased only in the inferior remission(IR) group (87). Accordingly, metabolome analysis showed that patients with SR, but not IR, were marked by elevated SCFAs including valerate and caproic acid (87). This study suggested that the beneficial response induced by cholestyramine was closely related to changes in intestinal symbiont composition and function (87). The addition of Lactobacillus rhamnosus GG (LGG) can inhibit the new synthesis of BA and increase the excretion of BA, thus preventing the liver damage and fibrosis induced by excessive BA in mice (88). In summary, gut microbiota can be used as a potential therapeutic target and diagnostic biomarker for PBC patients.

### Primary Sclerosing Cholangitis

Primary Sclerosing Cholangitis (PSC) is characterized by idiopathic intrahepatic and/or extrahepatic bile duct diffuse inflammation and fibrosis, leading to multifocal bile duct stenosis and chronic cholestasis (89). The pathogenesis of PSC is currently unclear; however, study (90) suggested that environmental and genetic factors contributed to its development. Among them, the intestinal flora has recently attracted attention as an environmental factor.

At present, there have been many researches on the changes of intestinal flora in PSC patients, and the specific changes of the flora have also been analyzed more clearly. Study (91) found an overall reduction in bacterial diversity in PSC patients compared to healthy states, with changes in the abundance of certain bacteria in the intestinal flora. Sabino et al. (53) reported that the feces of 66 Belgian PSC patients were overrepresented in the genera Enterococcus, Lactobacillus and Fusobacterium. Fukui (92) found an increase in fecal Haemophilus, Rothia, Clostridium, Enterococcus, Streptococcus, and Veillonella in 43 Czech PSC patients compared with healthy subjects. In another study, Nakamoto et al. (86) demonstrated that PSC patients exhibited bacterial dysbiosis.

Cholangiocytes are specialized cells arranged in the hepatic duct network. Cholangiocytes not only play roles in bile modification and transport,but also are capable of sensing endogenous and exogenous molecules, including PAMPs (93). This was also confirmed by a study (94) in which cultured cholangiocytes in PSC patients showed persistent hypersensitivity to LPS and other PAMPs. Cholangiocytes express many pathogen-recognition receptors, incorporating the NOD and all TLRs (95, 96). Autoantibodies against biliary cells were found in 63% of PSC patients. These antibodies can induce CD44 expression and proinflammatory IL-6 production in biliary cells, leading to enhanced damage and inflammation in biliary cell destruction (97). These autoantibodies include atypical perinuclear antineutrophil cytoplasmic antibody (p-ANCA), directed against the human autoantigen beta-tubulin isotype 5 (TBB-5) and so on, which is produced by nearly all commensal bacteria expression (98, 99). The combination of autoantibodies and biliary cells leads to rise in TLR expression, which in turn may sensitize biliary tract cells to microbial products. These receptors result in signaling cascades transduced through Myeloid differentiation factor88(MyD88)-dependent or independent pathways, and hepatobiliary inflammation and fibrosis can develop eventually (100). This is one of the possible mechanisms by which the intestinal flora results in the occurrence of PSC.

Furthermore, intestinal flora may also contribute to PSC/PSC-IBD nosogenesis through co-metabolism of synthetic compounds or host-produced molecules. For example, the BAs mentioned above. BAs acts as a key signaling molecule through interactions with several receptors, including the TGR5 and the FXR. These receptors play multiple roles the liver and gut (101). TGR5 agonists are involved in bile duct protection and anti-inflammatory effects; on cholangiocytes, TGR5 stimulates chloride secretion through cystic fibrosis transmembrane conductance regulator (CFTR) to maintain bicarbonate fimbriae; on macrophages, activation of TGR5 leads to a reduction in NF-κB transcriptional activity, which in turn reduces inflammatory cytokine production (102), while secondary BAs are the most potent TGR5 ligand. The study (103) showed that Germ-free mdr2−/− mice exhibited worsening biochemical and histological features of PSCs due to lack of secondary BAs, and increased senescence of cholangiocytes, and another study (83) uncovered that after 8 weeks of treatment with Colesevelam, the level of secondary BAs in Mdr2−/− mice was increased, and the liver and bile duct damage was reduced. Therefore, dysbiosis of secondary BAs metabolism caused by gut microbiota disturbance may be a potential key marker and mechanism of progressive biliary tract injury in PSCs. But so far, no complete and coherent causal pathway has been delineated between the micropopulation and clinical picture of PSC (101), more experiments are needed to verify these potential mechanisms.

At present, most clinical studies or animal experiments focus on exploring the relationship between PSC and intestinal flora dysbiosis, as well as the changes of intestinal flora in PSC patients or animal models. By far, most clinical studies targeting at gut microbiota were small-sample trials, multi-center and large-sample studies are urgently needed to provide higher-level evidence. Several small-sample clinical studies (104–106) with oral antibiotics have shown that the biomarker serum alkaline phosphatase (ALP) in PSC patients was significantly reduced by antibiotic treatment. Notably, a case report of oral vancomycin for the successful treatment of postoperative recurrent PSC in an orthotopic liver transplant patient (107). Another clinical study (108) involving 14 children with PSC showed that oral vancomycin was effective in the long-term treatment of children with sclerosing cholangitis, especially those without cirrhosis. This suggested that the pathogenic source of PSC might come from the recirculation of gut bacteria or the transmission of gut bacteria through blood. Relatively few studies have been conducted on probiotics for the treatment of PSC, and the results were controversial. A case reported (109) of a 13-year-old PSC patient receiving prednisolone, salazosulfapyridine, and probiotics (Lactobacillus casei Shirota)2 weeks later, the symptoms and laboratory indicators improved, the dose of prednisolone was tapered after subsequent treatments, and repeat biopsy 30 months later showed inflammatory cell infiltration and periductal fibers in each specimen. Significant improvement in fibrosis, fibrosis area decreased from 10.5 to 3.6%. FMT, as another therapeutic modality to intervene in the gut microbiota, has also been used in the treatment of PSC. A pilot clinical trial (110) showed that 30% of 10 PSCs received FMT, the ALP level decreased by ≥50%, and no adverse events occurred, proving the efficacy and safety of FMT in the treatment of PSC, but such studies were still relatively. The role of FMT remains to be further confirmed.

### Nonalcoholic Fatty Liver Disease

Nonalcoholic fatty liver disease (NAFLD) is a common clinical liver disease, which refers to the chronic metabolic diseases caused by diffuse fatty infiltration of hepatocytes caused by other factors except excessive drinking, including non-alcoholic fatty liver (NAFL) and nonalcoholic steatohepatitis (NASH), and related liver fibrosis. The disease can develop into liver cirrhosis and even hepatocellular carcinoma (HCC) without treatment (111). The occurrence of NAFLD is mostly caused by metabolic factors, such as excessive high-fat diet, more sitting and less exercise, and metabolic syndrome such as obesity, hypertension, hyperlipidemia, type 2 diabetes and so on (111). It is estimated that the global incidence of NAFLD is 25% and continues to rise globally in the context of the obesity epidemic (112). It has increasingly become a health problem that seriously endangers people’s health. At the beginning of the study of this disease, the researcher (113) put forward a hypothesis of “two strikes” to explain the pathogenesis of NAFLD. With the in-depth study of NAFLD in recent years, the theory of “second strike” has been difficult to fully explain the pathogenesis of NAFLD, and the theory of “multiple strikes” has gradually become a research hotspot. The theory holds that multiple pathological factors lead to the occurrence and progression of NAFLD, and different pathological factors also influence each other. In addition to insulin resistance, gut microbiota, inflammation, oxidative stress, genetic factors, dietary structure and other factors cause the occurrence of NAFLD (114). Among these risk factors, there is an increasing evidence that the gut-liver axis is involved in the development and progression of NAFLD. The related researches on intestinal flora and NAFLD are summarized as follows.

BAs are reabsorbed into the portal vein, where most molecules are captured by the liver, some remain in the bloodstream, where they act as signaling molecules through receptors such as TGR5 and FXR (115, 116). Activation of FXR increased fatty acid oxidation and decreased fatty acid uptake and synthesis (117). TGR5 activation increases energy expenditure and insulin sensitivity, while also increasing GLP-1 secretion, while GLP-1R agonists moderately reduce low density lipoproteins cholesterol((LDL-C), triglycerides, and total cholesterol (118). In NAFLD, bacterial abundance that convert primary BAs to secondary BAs decreases, so that both receptors are under-stimulated (44). As a result, the normal physiological functions of the two receptors cannot be performed normally, thereby aggravating NAFLD. Choline can facilitate the transport of very LDL from the liver, choline can be converted to trimethylamine (TMA) by the intestinal flora, and increased conversion of choline to TMA/TMAO in NAFLD leads to choline deficiency and accumulation of TMA/TMAO (119, 120), whereas choline deficiency leads to lipid accumulation in the liver, thereby exacerbating NAFLD.

In addition to affecting the metabolism of BAs and choline, the gut microbiota also produces metabolites, such as SCFA and endotoxins (LPS), which are also related to the pathological development of NAFLD. Studies have shown that SCFAs mediate modulation of intestinal flora and host inflammatory responses, for example, butyrate activates regulatory T cells in the gut, which in turn inhibit T cells and Th17 cells, thereby reducing pro-inflammatory signaling pathways (121, 122). Decreased levels of SCFAs production due to gut dysbiosis in NAFLD may contribute to a low-grade inflammatory state and reduce anti-inflammatory response capacity, while increasing gut permeability and increasing the risk of bacterial and LPS transfer to the systemic circulation (15). A lot of research have indicated that the LPS-TLR axis is in connection with the development of the NAFLD process (123). Animal experiments have shown that injecting mice to extremely low doses of LPS may significantly increase liver steatosis (124), and a clinical study found that compared with healthy controls, obese patients had higher levels of LPS and related biomarkers (such as TLR2 and TLR4) at a higher level (125). At the molecular level, LPS is a PAMP recognized by specific PRRs called TLR4 and its co-receptors LPS-binding protein and CD14. Activation of TLR4 triggers a downstream inflammatory cascade (126, 127). LPS can stimulate TLR4 on HSCs, Kupffer cells or hepatocytes, triggering an innate immune response that causes inflammation throughout the body (128) (129, 130).

Given the critical role of intestinal flora biota dysplasia in NAFLD pathogenesis, significant progress has been made to explore ways in which gut microbiota biota biodistribution and elucidated metabolites can be corrected. These methods include the use of probiotics, prebiotics and synthetic bacteria, as well as the use of fecal microbiota transplantation(FMT).These intervention modes have been widely studied in clinical trials and animal models and achieved good results. Various probiotics were tested in NAFLD animal models. And the results obtained are promising (131). Lactobacillus plantarum has been found to improve NAFLD (132). Another probiotic species, LGG, was able to reduce liver fat accumulation and hepatic inflammation (133). In addition, there are studies using combinations of various bacteria to improve probiotic efficacy. VSL#3 is a formulation of 8 bacterial strains including Bifidobacterium, Streptococcus thermophilus, and so on. Results from animal experiments (134) and clinical studies (135) have demonstrated that it inhibits NF-kB, reduces liver inflammation, reduces liver fat accumulation, and improves insulin sensitivity. Probiotics are an indigestible food ingredient that promotes the growth and activity of specific or few microbiota in the host gut (136). For example, studies have shown that fructooligosaccharides (FOS) can restore intestinal epithelial barrier function in NAFLD mice fed a methionine-choline deficient diet and improve liver steatosis and inflammation (137, 138). Inulin is another probiotic with positive effects on lipid metabolism. lipid. scholars (139) reported that in inulin fed mice, SCFA production increased, and the expression of genes involved in adipogenesis and fatty acid longation/unsaturated also reduced. Synbiotics are a combination of probiotics and prebiotics (140). Synbiotics reduced hepatic steatosis and insulin resistance in a NAFLD rat model on a high fructose diet (141). The results of a clinical study (142) conducted by Malaguarnera et al. showed that synbiotics constituted of Bifidobacterium longum and FOS reduced aspartate aminotransferase, serum endotoxin levels, cholesterol, and the degree of hepatic steatosis in NAFLD patients. FMT is another therapeutic modality that targets the gut microbiota. Compared with synbiotics and probiotics, FMT can provide a broad range of symbiotic bacteria and other microorganisms to promote the maintenance of intestinal microbial ecology. Some animal experiments show that FMT has some effect on the treatment of NASH. Zhou et al. (143) found that after 8 weeks of FMT treatment, beneficial bacteria Lactobacillus and Christensenellaceae, butyrate concentrations increased, endotoxemia and intrahepatic lipids in high-fiber diet-fed mice accumulation has dropped. Vrieze et al. (144)reported that FMT increased insulin sensitivity in patients from healthy lean individuals to those with metabolic syndrome. It also improved the intestinal flora by increasing the number of bacteria that produce butyrate in the gut.

The intestinal flora is involved in NAFLD. Different stages of NAFLD have different characteristics with intestinal flora (145).. Changes in intestinal flora diversity and abundance are mediated by multiple bacterial metabolites, including BAs, choline, SCFA, and LPS. Some animal experiments and intestinal flora regulation, as well as supplementation with bacterial metabolites, may have therapeutic effects.

### Cirrhosis and Its Complications

Cirrhosis is the terminal stage during the development of various chronic liver diseases and is widely prevalent worldwide. It is caused by heavy alcohol consumption, obesity, NAFLD, viral infections, cholestatic diseases, AIDs, and copper or iron overload (146). As the disease progresses, portal hypertension, infection, hepatic encephalopathy, ascites and other complications can affect the prognosis of patients with cirrhosis and increase the fatality rate. As early as the middle of the 20th century, some scholars have proposed that Lactobacillus acidophilus can change the intestinal flora to treat hepatic encephalopathy (147). Later, scholars gradually found that patients with cirrhosis in the small intestine bacteria overgrowth, fecal bacterial composition is abnormal. Cirrhosis patients tend to have varying degrees of imbalance in their gut microbiota, mainly in proportion, type, quantity and metabolic activity of gut microbiota, and changes in the local distribution of intestinal microbiota. In 2011, Chinese academician Li Lanjuan’s team first revealed the characteristics of fecal flora of patients with cirrhosis (148). At the phylum level, Proteobacteria and Fusobacteria rised significantly, but Bacteroidetes numbers were decreased in patients with cirrhosis (148). In terms of family level, the number of Lachnospiraceae decreased significantly in cirrhosis patients, while the number of Enterobacter, Streptococcus and Veillonellaceae increased significantly (148). In 2014, some scholars used metagenomic technology and a larger population to analyze the characteristics of the disease flora associated with cirrhosis, and obtained 15 biomarkers, which can be used to accurately predict cirrhosis (149). In addition, it has been proposed that the biological dysregulation ratio of cirrhosis may be a useful quantitative indicator for describing changes in the microbiota associated with cirrhosis progression (150).

On the one hand, cirrhosis can cause intestinal microbiota changes (151), on the other hand, intestinal microbiota imbalance can promote the progression of cirrhosis. Some scholars discovered that the recto-sigmoid mucosa-microbiota showed higher potential pathogens and lower autochthonous bacterial abundance in cirrhosis (152). In patients with cirrhosis, intestinal mucosal permeability is enhanced, and a large amount of LPS is generated when the cell wall disintegrates after the death of Gram-negative bacteria, which is absorbed by the intestine and then enters the liver, leading to inflammation through the activation of TLR signaling pathway (153). Teltschik et al. (154)demonstrated that the reduction of antimicrobial peptides secreted by panth cells in the intestine of cirrhotic rats was most obvious in the cecum and ileum, where most bacterial translocations occurred. Damage to the anti-microbial mechanisms of cirrhosis may exacerbate bacterial translocation. However, Pérez-Paramo et al. found that bacterial translocation only occurred in rats with both increased intestinal mucosal permeability and intestinal bacterial overgrowth (155). Animal experiments confirmed that no small intestinal bacterial overgrowth of cirrhosis rats generally did not happen bacterial translocation, eventually transferred to the mesenteric lymph nodes of bacteria and excessive growth of bacteria in the gut bacteria tend to be the same (155).The gut microbiome is unbalanced, causing damage to intestinal mucosal barrier damage and excessive intestinal endotoxin to circulate through the portal vein into the liver and throughout the body. Increased levels of endotoxin and pro-inflammatory cytokines stimulate the activation and proliferation of HSCs, secrete a large amount of extracellular matrix, and promote the proliferation and deposition of intrahepatic fibrous connective tissue, and promote the development of cirrhosis. LPS of the liver can increase intrahepatic resistance and visceral blood flow, thus aggravating portal hypertension (156).

Hepatic encephalopathy(HE) is a syndrome caused by chronic or acute liver failure, decompensated cirrhosis, or various abnormal portal veno-systemic circulation shunt. HE manifests itself in a wide range of neuropsychiatric disorders, from mild cognitive impairment to significant disorientation, delirium and coma (157). Some gut bacteria produced blood ammonia and pseudo-neurotransmitters, study suggested (158), and intestinal flora plays a crucial part in the enterohepatic circulation of urea. Ammonia produced by various intestinal bacteria and their metabolites is a vital source of ammonia in the human intestine, such as urease-producing bacteria Klebsiella, Proteus and Helicobacter pylori (159, 160). Zhang et al. found that contrasted with healthy control population, Prevotella and Enterobacter were increased in patients with mild and miniature HE, while Eubacter and Sutrella were absent (161). Bajaj et al. found that the increase of porphyromonas belonging to Bacteroides may be related to the occurrence of HE (152). When intestinal microecology was disturbed, urease producing bacteria overgrew and ammonia production increased. The number of beneficial bacteria producing acid is reduced, the dissolution and elimination of ammonia are reduced, leading to hyperammoniasis under the common action, and then can cause the occurrence of HE. Toxins such as ammonia and inflammatory cytokines, produced by the damaged gut environment, enter the bloodstream and worsen or precipitate HE (162).

Ascites is one of the common complications in the late stage of liver disease and an important marker of disease progression. The case fatality rate of 1 year after the occurrence of ascites is about 15%, and that of 5 years is up to 44%-85% (163, 164). Goelz H et al. used the second-generation and third-generation sequencing methods and found that 33 (66%) ascites samples contained Enterobacter faecalis and Klebsiella, and 28 (56%) ascites samples contained anaerobic bacteria (165). Tuomisto et al. have shown that the number of Gram-negative enterobacter in faeces of patients with alcoholic cirrhosis increases, and enterobacter DNA was detected in ascites up to 50% of patients without spontaneous bacterial peritonitis (166). Shamsaddini et al. (167) showed that Pseudomonas, Serratia and Clostridium perfringens were increasing in samples of patients with ascites. Increased intestinal mucosal permeability, dysregulation of gut microbiota, and migration of bacteria and their PAMPs further promote the progression of liver disease through activation and induction of pro-inflammatory states by the immune system (168). Feng Y et al. (169)found that the plasma inflammatory factor content in model group was significantly higher than that in control group, and significantly lower than that in probiotic intervention group. The inflammatory response to the release of proinflammatory cytokines increased the production of intraarterial nitric oxide, which exacerbated visceral vasodilation and insufficiency of effective arteries, and exacerbated water and sodium retention and ascites formation (170, 171). LPS induced endotoxin tolerance through the TLR-4 dependent pathway, which was characterized by inhibition of antigen presentation, reduction of pro-inflammatory mediators, and overexpression of anti-inflammatory signaling molecules (172). Microecologics such as lactobacillus mixture inhibited oxidative stress and inflammation by modulating the TLR-4/NF-κB signaling pathway, while lactobacillus mixture reduced Gram-negative bacteria and LPS entry into the portal vein, thereby alleviating liver damage (173).

Spontaneous bacterial perionitis (SBP) is a peritoneal infection caused by pathogenic bacteria passing through the intestinal, blood or lymphatic system in the absence of definite intraperitoneal disease based on cirrhosis, and is a common complication and cause of death in patients with end-stage liver diseases. The most common symptoms of SBP are chills, fever and abdominal pain. The most common infection source of SBP is Gram-negative bacteria, and intestinal bacterial translocation plays a crucial part in the occurrence and development of SBP. SBP occurs when bacteria normally living in the gut enter the abdominal cavity and the ascites become infected. This occurs in advanced liver disease because of a weakened immune system response and changes in the bacterial environment in the gut (174). Enhanced intestinal permeability in cirrhosis promotes systemic endotoxemia and plays a vital role in the pathogenesis of SBP. The intestinal epithelial tight junction barrier protects against a wide variety of intestinal microorganisms and regulates passive extracellular penetration of various water-soluble molecules and bacterial antigens. Assimakopoulos et al. proved for the first time that intestinal tight junction proteins in cirrhotic patients were significantly altered (175).

Conventional treatment of cirrhosis focuses on the treatment of causes and complications, and in some cases may require a cases liver transplantation. Correcting gut microbiome imbalance can alter the progression of cirrhosis (176). Antibiotics can eliminate intestinal pathogens, and rifaximin is a non-absorbable antibiotic. Rifaximin can promote the growth of bifidobacteria, lactic acid bacteria and other beneficial bacteria (177). Study showed that long-term use of rifaximin improved outcomes in patients with decompensated alcoholic cirrhosis (178). Feng et al. found that the liver lobule structure was significantly improved, edema cells were significantly reduced, collagen fiber proliferation was significantly reduced, and the degree of cirrhosis was significantly reduced in the probiotic group compared with the model group (169). Horvath et al. showed that the supplementation of multiple probiotics could enrich the probiotics in patients with compensatory cirrhosis, and effectively change the probiotics and intestinal barrier function (179). Fluoroquinolones reduced the risk of first episode and mortality of SBP in patients with cirrhosis, and Rifaximin prevented and relieved overt encephalopathy (180). A real world study analyzed the changes in intestinal flora characteristics before and after rifaximin treatment for refractory ascites, and the results showed that the abundance of Haemophilus, Roxella and Prevota in the rifaximin group was significantly reduced, confirming that rifaximin could regulate the structure and function of gut bacteria, then improving systemic inflammation (181). Studies have shown that Bacillus clausii UBBC07 and Lactobacillus plantarum UBLP40 can reduce hyperammonemia and intestinal bacterial overgrowth, thereby preventing HE development (182).

### Liver Cancer

Liver cancer generally progresses from chronic liver diseases(CLD), and most types of liver diseases have a potential risk of developing liver cancer. Alterations in intestinal permeability and intestinal microbiota composition are prominent features of all sorts of end-stage CLDs, and also appear in the initial stage of some CLDs. Hence the gut microbiota can affect the progression of CLDs at different periods and may give contribution to the progress of liver cancer in all of these periods. As mentioned earlier, the gut-liver axis is the way in which the intestinal flora affects the liver.

Recently, increasing evidence suggested that disruption of the gut-liver axis contributed to the development of most kinds of CLDs, involving liver cirrhosis and liver cancer (183). The gut-liver axis boosts liver cancer progress through the two key mechanisms, leaky gut and microbiota dysbiosis (184). “Leaky gut” refers to a condition in which the intestinal permeability of the microbiota and its metabolites is increased. The mechanism of leaky gut is multifactorial, for example, alcohol consumption or high-fat diet can disrupt the intestinal flora and disrupt the gut barrier resulting in leaky gut (184). As studies have shown, by changing the expression of tight junction proteins zonula occludens-1 (ZO-1) and claudin-1, alcohol can enhance the permeability of gut (185, 186). Leaky gut allows MAMPs and bacterial metabolites to translocate more easily and reach the liver. MAMPs such as LTA and LPS, have been proved to result in liver inflammation through irritating TLR4 (187, 188), TLR9 (189) and TLR2 (190, 191). TLR4 is expressed in different kinds of hepatic cells, including HSCs, hepatocytes, besides Kupffer cells. LPS can be recognized by TLR4 and bind to produce a response (192). Animal experiments have shown that the hepatic cells we mentioned above that assembled with TLR4, were responsible for promoting fibrogenesis or carcinogenesis (193). Epiregulin, a member of the epiregulin family, has potent mitogenic effects on hepatocytes (194), and study (193) have found that epiregulin-deficient mice reduced hepatocarcinogenesis, while activating TLR4 caused the NF-κB-mediated hepatocyte lysis. The proto-up-regulated protein was up-regulated13, suggesting that a key mechanism by which the LPS-TLR4 axis promotes hepatocarcinogenesis is the prevention of hepatocyte apoptosis mediated by the NF-κB signaling pathway. Moreover, activation of TLR4 by LPS in liver cancer cell lines enhanced their invasive potential and induced epithelial-mesenchymal transition (195). Lipoic acid, a bacterial ingredient of the gut, acts as a ligand for TLR2 and promotes hepatocellular carcinoma development in obese mice by increasing tumor-promoting senescence-associated secretory phenotype (SASP) and COX2 expression in HSCs (184). These findings suggested that leaky gut promoted hepatocarcinogenesis through MAMP-TLR-mediated signaling pathway.

Microbiota dysbiosis alters the metabolic pathways of the gut microbiota (196), thereby affecting various pathological process of the progress of CLDs and liver cancer. In an animal experiment, changes in gut microbiota profiles resulted in increased deoxycholic acid in the enterohepatic circulation in a mouse model of liver cancer, which increased the secretion of senescence-related phenotypes in HSCs, leading to various inflammations and tumor-promoting factor secretion, and promoted the development of liver cancer (197). Genes expressed by gut microbes ferment the sugar in food into ethanol, which is absorbed by the gastrointestinal tract through simple diffusion (198). Ethanol, a known carcinogen, also exacerbates oxidative stress and liver inflammation (199). However, whether endogenous ethanol produced by the intestinal flora promotes the progression of CLD and contributes to the development of liver cancer has not yet been identified and needs to be further investigated.

There are a large number of immune cells in the liver, and there was experimental evidence that innate or adaptive immune responses can promote or inhibit the progress of liver cancer (200). Part of the reason may be that liver cancer often develops in the context of chronic inflammation. Although immune cells are crucial players in immune surveillance, they may also contribute to carcinogenesis in CLDs by promoting inflammation (196). Under the stimulation of endotoxin, monocytes in the blood circulation can penetrate into liver tissue and differentiate into macrophages, thus increasing the production of inflammatory mediators and oxygen free radicals, leading to liver lesion or even hepatic failure (201). Gut microbiota dysbiosis induces IL-25 production and promotes liver cancer progression by activating macrophage replacement and CXCL-10 secretion in the tumor microenvironment (202). But on the other hand, the gut microbiota modulates the chemokine CXCL16 levels in hepatic sinusoidal endothelial cells (LSEC) by regarding BAs as messengers, thus controlling the accumulation of CXCR6+ NKT cells in liver. Accumulated NKT cells have an activated phenotype that inhibits liver tumor growth (203). Based on the above studies, gut microbes may modulate disease progression in HCC patients in positive or negative ways.

Since gut flora has been found to be a vital role in promoting the progress of CLDs and liver cancer in animal experiments, it is easy to consider the intestinal flora as an important target for liver cancer treatment (196). Various methods are currently available such as antibiotics, probiotics etc. Animal study (204) indicated that the use of antibiotics in advanced treatment may help palliate the development of liver cancer. Continuous intestinal disinfection with an oral antibiotic cocktail consisting of ampicillin, neomycin, metronidazole, and vancomycin was shown to effectively reduce the number and size of liver cancer in mice (193, 197). Probiotics have been proposed to rebalance gut microbiota in CLDs by promoting the recovery of beneficial microorganism. For example, administration of VSL#3 alleviates intestinal dysplasia, reduces intestinal inflammation, and inhibits liver tumor growth and diversity in a DEN-induced rat model of hepatocarcinogenesis (205). Another study (206) reported that the probiotic cocktail Prohep reduced the amount of Th17 cells in tumors, thereby inhibiting the progress of liver cancer in model mice. BAs are significant regulators of intestinal parclose, and FXR is a type of receptor for BAs, mediating the multiple effects of BAs on liver metabolism, such as inhibiting the synthesis of BAs, liver inflammation and tumor growth, etc. (207). Study (208) showed that mice deficient in FXR had impaired gut integrity and increased incidence of liver cancer. GW4064 or Obeticholic acid (OCA) as FXR agonists can alleviate the lesion of intestinal mucosa, reduce the permeability of gut parclose, restrict bacterial overgrowth and bacterial translocation in model animals (209–211), suggesting that FXR agonists may have a positive effect on the prevention of liver cancer effect. Postbiotics are the metabolites of gut microbiota, including SCFAs, bacteriocins, polysaccharides, vitamins, etc., which can prompt the recovery of the function of gut parclose (212). Research have indicated that postbiotics have anti-proliferative and anti-cancer characters that can regulate the curative effect and ameliorate the side effects of the existing therapy, and thus act as a promising method for auxiliary treatment for liver cancer (213). A retrospective research (214) reported that propranolol might decrease the morbidity of liver cancer in patients with hepatitis C-related cirrhosis, suggesting a potential role for liver cancer prevention. Although in animal models, the prokinetic cisapride can not only shorten the time of gut transportation, but inhibit the overgrowth of gut flora and bacterial translocation (215), the long-term benefits for CLD patients, especially patients with liver cancer, need to be further studied.

Above we have concluded how the gut microbiota affects liver cancer through leaky gut and dysbiosis, and also introduced potential treatments aiming at gut microbiota for liver cancer. Clinical studies, especially randomized controlled studies, and the specific pharmacological mechanism are still lack of investment, so further exploration is urgently needed.

## Conclusion and Prospect

Gut microbiota is usually considered to be closely related to metabolic diseases. Since our human body is an organic whole, exploring this vital symbiotic ecosystem will surely lead us to further comprehension to various diseases. At the same time, the intestinal flora can also be regarded as a corresponding therapeutic target, providing another perspective for the development of clinical pharmacy. Therefore, it is of great significance to clarify the role of the intestinal flora in diseases of different systems. In this article, we reviewed the role of the gut microbiota in various liver diseases and interventions that target at it for therapy. In general, the liver and the intestine have a close connection through the gut-liver axis. The destruction of the intestinal barrier and the imbalance of the gut microbiota are the initiating factors that cause hepatic lesions. In different kinds or stages of hepatopathy, the composition of the gut microbiota is disparate. LPS, SCFAs, BA, and other bacterial metabolites are the main signals inducing immune responses in liver tissue. The TLR family plays a key role in mediating these signals to produce liver inflammation. In terms of treatment, antibiotics, probiotics, prebiotics, synbiotics, and fecal microbiota transplantation are the main therapeutic methods targeting at gut microbiota, and their effectiveness has been confirmed in animal experiments and some small clinical studies.

However, current researches on the gut microbiota and liver diseases also face the following challenges. First of all, most of these researches are animal experiments, which aim to explain the potential correlation between gut microbiota and liver diseases, and few of them can form an exact and coherent pathological mechanism. Therefore, more in-depth human researches are urgently needed to verify these potential mechanisms. Secondly, the intestinal flora is susceptible to genetic, environmental, behavioral and other factors, and further multi-racial, long-term, multicenter studies and better matching controls are needed to explore the association between the intestinal flora and specific diseases.

The diversity of gut flora and the differences between individuals make it important to look for core flora that really works. With the development of sequencing technology and the revolution in metagenomics, enterotype have been proposed to characterize the intestinal flora (216). Through the analysis of the enterotype, the characteristics of core flora will be extracted to discover more potential next-generation probiotics(NGPs). In the future, the efficacy and safety of the NGPs may be tested through clinical studies. And we may see NGPs developed for specific health problems, enabling customized probiotics for patients.

## Author Contributions

All the authors participated with suggestions and the development of this manuscript. LW and ZC participated in drafting the article and/or revising it critically for important intellectual content, and also created the figures and tables. WL and LZ participated in the study conception, critical revision of the article, and supervision. JL has made great contributions to the revision process of the article.

## Funding

National Key Research and Development Program of China (No. 2018YFC1705700).

## Conflict of Interest

The authors declare that the research was conducted in the absence of any commercial or financial relationships that could be construed as a potential conflict of interest.

## Publisher’s Note

All claims expressed in this article are solely those of the authors and do not necessarily represent those of their affiliated organizations, or those of the publisher, the editors and the reviewers. Any product that may be evaluated in this article, or claim that may be made by its manufacturer, is not guaranteed or endorsed by the publisher.
